# Collaborative mentoring for effective medical research groups

**DOI:** 10.15694/mep.2019.000214.1

**Published:** 2019-11-26

**Authors:** Rodrigo Enrique Elizondo-Omaña, Pablo Patricio Zarate-Garza, Guillermo Jacobo-Baca, Yolanda Salinas-Alvarez, Bernardo Alfonso Fernandez-Rodarte, Javier Humberto Martinez-Garza, Alejandro Quiroga-Garza, Santos Guzman-Lopez

**Affiliations:** 1 Universidad Autonoma de Nuevo Leon

**Keywords:** mentoring, collaborative mentoring, academic medicine, medical research group, anatomy research group, undergraduate students, research mentoring.

## Abstract

This article was migrated. The article was marked as recommended.

Research benefits professors and students, mentors and mentees, however, many Universities lack formal programs, especially in basic sciences such as anatomy. Faculty, many times, lack the time, resources, and a well-structured program. Mentoring and collaborative work, have played an important role in creating an effective environment that inspires its members into scientific production, enhances research skills, while gaining experience. The authors presented a student anatomy research group (Grupo de Investigación en Anatomía [GIA]) model that integrates faculty from basic and clinical fields through a collaborative mentoring and tightly organized structure that increases training, experience, and scientific output. The transformation stages described shows the progress, reporting the fundamental elements for integration of the model, with results presented at 16 years of experience of the group in the Anatomy department. Results evidence a steady increase in student/professor involvement, scientific publishing, presentations in meetings (national/international), and cites.

## Introduction

The benefits of mentoring students and scientific research have been widely described ( Brown, Daly and Leong, 2009; Ratnapalan, 2010; Palmer *et al*., 2015; Taype Rondán *et al*., 2015; Tan *et al*., 2018; Havard & Baker, 2018) however, the effectiveness of the diverse existing models used for undergraduate medical research is limited ( Pololi and Knight 2005; Linn *et al*., 2015; Castejón Cruz, 2014; Quispe-Juli *et al*., 2019). Most authors agree that research is an important characteristic of any faculty member. Chopin, S.F. would even argue a good researcher with poor teaching skills surpasses a great teacher with low or no research output, in terms of value for a University, and professional opportunities, such as promotions ( Chopin, 2002; Mullen, 2000). Because of this, many young faculty members seek to form part of research activities, and in order to accelerate the process, it is important to collaborate and include students (undergraduate, graduate, post-graduate) to enhance productivity ( Pololi *et al*., 2002; Brown, Daly and Leong, 2009; Feldman, Divoll and Rogan-Klyve, 2013; Menon, Muraleedharan and Bhat, 2016; Havard and Baker, 2018). Activities may include journal clubs, research groups, and collaboration with more experienced researchers or colleagues ( Desai *et al*., 2008; Mullen, 2000; Linn *et al*., 2015; Stevenson *et al*., 2017; O’Loughlin, Husmann and Brokaw, 2018).

When two or more individuals from different educational levels/expertise come together to work on a common objective, a certain grade of mentoring will naturally evolve. A basic fundament of any University. A mentor plays an important role in inspiring mentees, having a significant impact on them professionally and personally ( Hauer *et al*., 2005; Brown, Daly and Leong, 2009; Chopin, 2002; Gershenfeld, 2014; Linn *et al*., 2015; Palmer *et al*., 2015; O’Loughlin, Husmann and Brokaw, 2018). Mentoring has many forms and aspects, but it also demands a substantial amount of time and commitment with most benefits in favor of the mentee, causing a decline of research mentors ( Pololi and Knight, 2005; Brown, Daly and Leong, 2009; Ratnapalan, 2010; Menon, Muraleedharan and Bhat, 2016). In order for a research mentoring to succeed, several components need to be evaluated: 1) Mentor-mentee ratio, 2) Assignment (required vs voluntary), 3) Compensation for mentor and mentee, 4) Frequency of meetings and duration of mentorship, and 5) Structure (structure and resources) ( Gershenfeld, 2014; Tan *et al*., 2018).

Many studies and reviews agree a low mentor-mentee ratio has a better outcome, although a one-on-one method is impractical. Most mentors have from 1 to 3 students as mentees, however few studies have reported positive results in high ratio programs (up to 30 mentees) or with multiple/collaborative mentors ( Desai *et al*., 2008; Feldman, Divoll and Rogan-Klyve, 2013; Amgad *et al*., 2015; Schwartz *et al*., 2016; Stevenson *et al*., 2017; Tan *et al*., 2018), although mentor availability may not always be the case. When either party is assigned, the relationship may be forced, and although productive, many of the psychosocial aspects of the mentoring will not develop. A voluntary relationship where both, mentor and mentee choose each other, develops a more compatible working environment, as they share mutual interests. Financial compensation for mentors is rarely the case ( Gershenfeld, 2014; Tan *et al*., 2018). The benefits are usually based on self-satisfaction and professional growth for the mentor. The mentee invests time as an extracurricular activity, but in return gains academic knowledge, opportunities to present in meetings, and sometimes authorship to a scientific paper, encouraging future engagements in research. Many times, they also develop an identity by integrating behaviors, attitudes, and values of their mentors and those around them. Skills learned include: enhanced research, critical thinking, effective work, professionalism, peer-collaboration, experience reading, discussing and reviewing papers, analyzing data, and writing manuscripts ( Brown, Daly and Leong, 2009; Pololi *et al*., 2002; Feldman, Divoll and Rogan-Klyve, 2013; Palmer *et al*., 2015). The frequency and length of meetings vary on the objectives and environment, as well as the duration of the programs ( Menon, Muraleedharan and Bhat, 2016; Tan *et al*., 2018). Most reviews are unable to compare this variable to performance due to a lack of time period or transverse reports. However, tightly organized and structured programs with adequate supervision tend to have better outcomes, resources, training, and consistency ( Feldman, Divoll and Rogan-Klyve, 2013; Tan *et al*., 2018).

The authors present an undergraduate medical student anatomy research group based on a collaborative mentoring with a tightly organized and well-structured model with results at 16 years.

## Description

**Figure 1.**
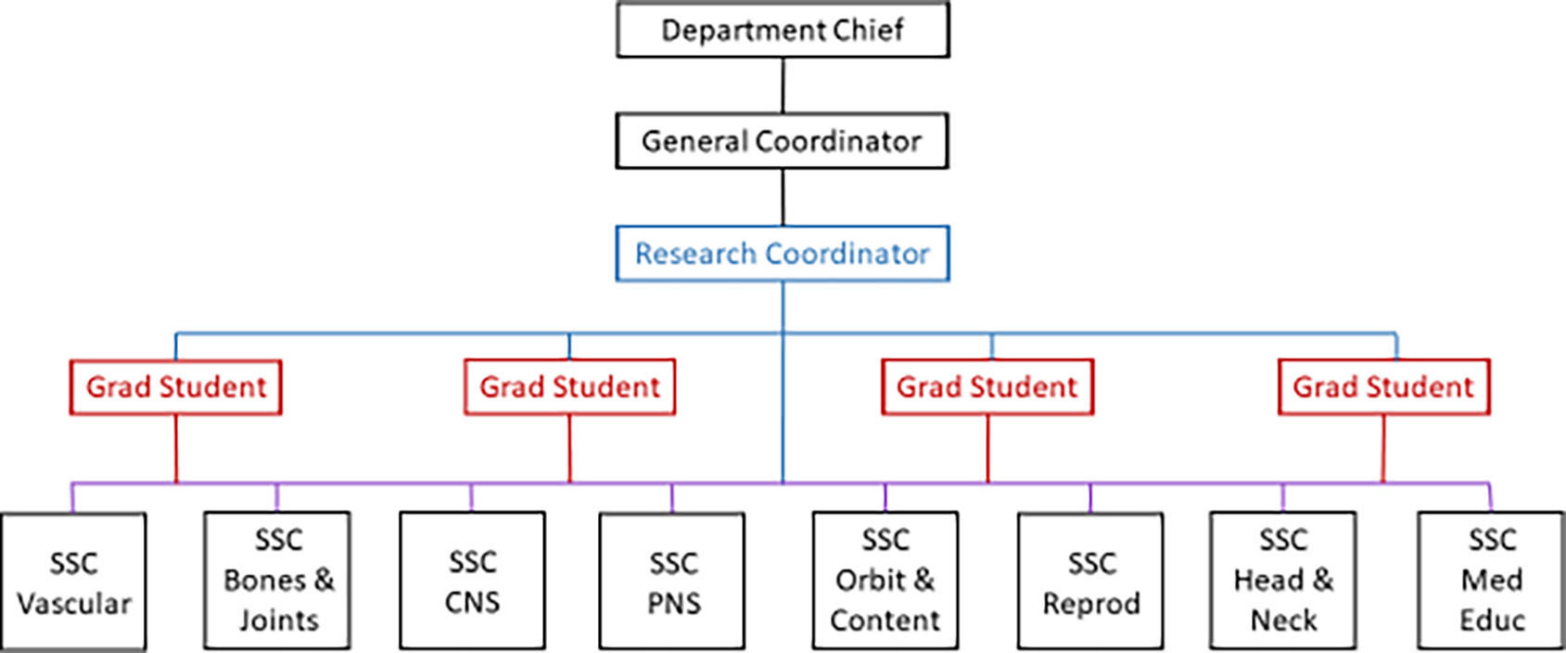
Anatomy Research Group Structure and communication flow.

**Figure 2.**
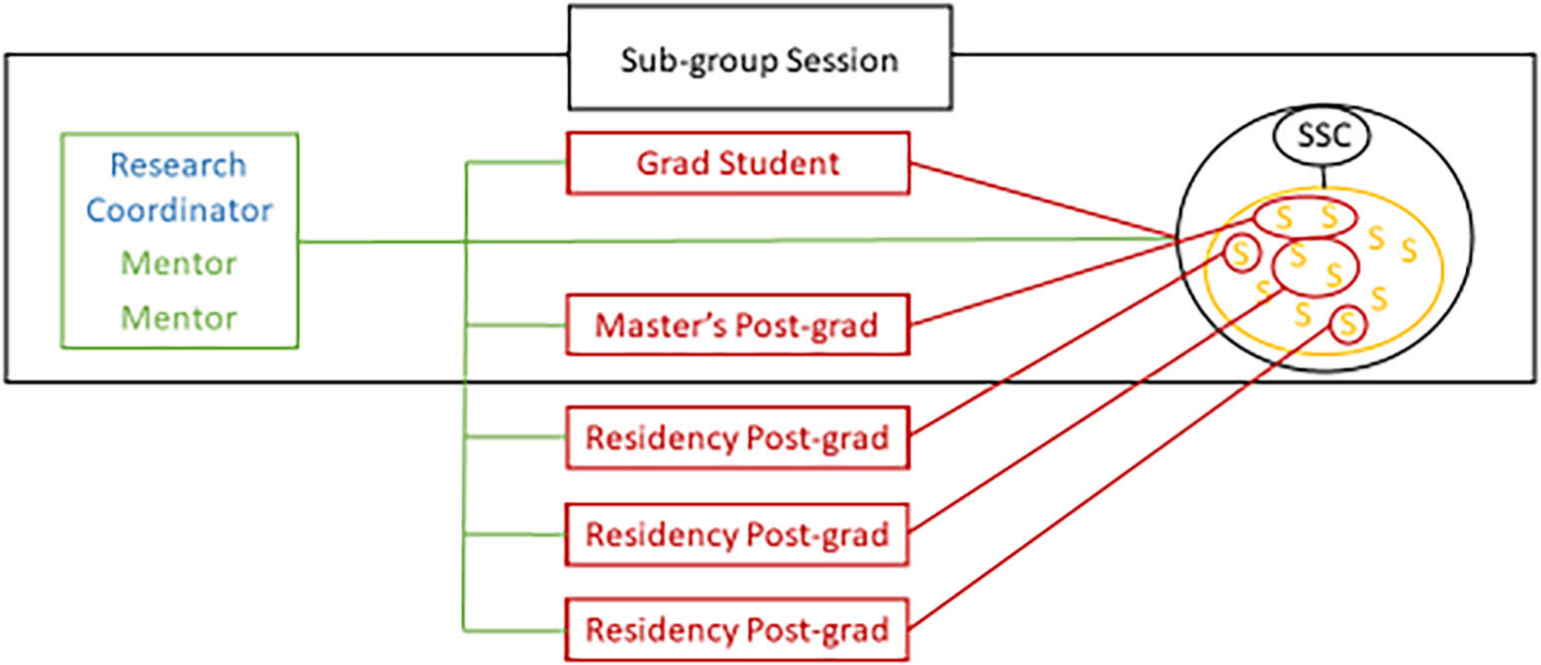
Anatomy Research Sub-Group Session Structure and communication flow.

**Model Development** The model development was set in the anatomy department from the medical school based in the public state university, Universidad Autónoma de Nuevo León (UANL) in Monterrey, Mexico. The department had no prior research experience, was based on traditional memorization teaching, had limited resources, and a large volume of students (>500 students per semester). The Anatomy Research Group ( *Grupo de Investigación en Anatomía* [GIA]) was officially founded in 2003 (Morelos-Avalos, Elizondo-Omana and Guzman-Lopez, 2014; Quiroga-Garza, Elizondo-Omana and Guzman-Lopez, 2018). The objective was a long-term plan to include morphology research and transform the teaching methods of the department (the latter will not be discussed in this paper). This was accomplished by incorporating young, motivated, faculty members with interest in research, and committed to forming a journal club with the objective of publishing. The group has evolved from a loosely organized group with a low frequency of meetings and a low number of mentors, to a well-structured, tightly organized ( Figure 1), collaborative mentoring ( Figure 2) group during a four-stage process.

Department Chief works primarily with the General Coordinator, as well as all professors. The General Coordinator administrates the different departmental coordinators, in this case, oversees all research activities through the Research Coordinator with several meetings per week. The Research Coordinator has weekly meetings with Grad Students, monthly meetings with SSC, and a variation of meetings with individual mentees. Grad Students help coordinate all activities by having weekly updates from the SSC and students from their respective subgroups. Grad Student: Graduate student; SSC: Student subgroup coordinator; CNS: central nervous system; PNS: peripheral nervous system; Reprod: Reproductive; Med Educ: Medical Education.

Research Coordinator acts as a mentor in the subgroup. Graduate students and Master’s post-graduate students attend meetings with the students. Residency post-graduate students are medical residents from different branches, according to the subgroup, and work with mentors and students outside meetings. Grad Student: Graduate student; Post-grad: post-graduate student; SSC: Student subgroup coordinator.

### Stage 1 (2003-2005)

One professor and five students. It began as an extra-curricular activity during summer break and an introduction into research with theory classes. After the summer, students with initiative continued to individually search for the professor as a mentor to collaborate in activities of a project. Meetings were irregular. Language for publication was a limitation (English as a second language), and insufficient experience with scientific writing. **Output:** Publication of basic scientific work in local or national journals in Spanish. Undergraduate students not always included in the paper as authors.

### Stage 2 (2005-2010)

The group transformed into a journal club, allowing between 8 and 12 medical students to join. Professors from the Embryology and Histology Departments were recruited. Postgraduate students from the masters and doctorate program began participating. Weekly one-hour sessions were established during the semester to discuss papers. Research training courses and workshops were also implemented. Students were encouraged to propose an idea based on the journal club discussion, the professors refine, direct, focus the idea, and provide access and materials to execute. Collaboration was established with other schools within the university. **Output:** Members are better trained; students begin to publish as first authors; experience through student participation with posters or oral presentations in local or national research and/or anatomical meetings; well-structured publications (2-4/year) in indexed journals with international reach.

### Stage 3 (2010-2014)

Collaborative mentoring was implemented. The journal club was split into a general group for introduction and five topic-oriented subgroups, (vascular system [2010], bones & joints [2010], central nervous system [2011], peripheral nervous system [2011], orbit & content [2011]) maintaining an 8 to 12 student ratio per subgroup. Professors from clinical fields, as well as postgraduate students from medical residencies (Radiology, Orthopedics & Traumatology, Sports & Rehabilitation, Neurosurgery, Neurology, Plastic Surgery, General Surgery, Ophthalmology) were involved in the corresponding subgroups. Students were still responsible for generating the idea based on the weekly meetings, with 2 to 4 professors/mentors guiding the discussion of ideas and papers with the rest of the group. Collaboration was established with other national universities. Group regulations were established in a structured programmatic handbook defining the roles and responsibilities of all members. **Output:** Subject of interest-oriented workshops are implemented every year in each subgroup; critical reading and writing courses are formalized and organized yearly; students continue as first authors in their publications; student participation increases in national meetings, primarily with oral presentations, if not, posters; indexed journals publication is maintained with increase in project registration.

### Stage 4 (2014-Present)

The journal club continued to grow, with an increase in student interest and participation. Three more subgroups were created (reproductive system [2014], head & neck [2015], medical education [2017]) with the same student ratio, adding professors and residents from other clinical departments (Gynecology & Obstetrics, Otorhinolaryngology, Education/Academic Medicine). International collaboration with other Universities is established. Research students are aided in international research training and participation. **Output:** Research-experienced students with 1 or more published papers as a first or second author; postgraduate students with internationally obtained masters or doctorate degrees; participation increase in national and international meetings; indexed journals publication upturns with steady increase; cite score increases demonstrating the relevance of publications; English is no longer a limitation.

### Group Characteristics and Structure

To construct an effective research group, three elements were essential: student initiative, persistence, and collaborative mentoring.

The student initiative is key. Participation is voluntary, no student has been forced or pressured into the program. Those who are interested may register. There is no minimum GPA or research background required.

Persistence is the main requirement for students who get involved. We provide a semester course with a weekly two-hour meeting to discuss the fundamentals of research and exercises. The student may also incorporate into a research summer (4 weeks, 6 hours/day, Monday-Friday) and if attendance is ≥80% they can join one of the eight GIA subgroups.

Collaborative mentoring has also been crucial for the success of the program. Each subgroup has 2 and 4 professors that act as mentors. Students are not assigned to a particular mentor and may work freely between several of them. Most mentors are also clinicians, so emergency calls may occur. The presence of several professors allows for consistency, good interaction with all members, and avoids canceling meetings when a mentor is absent due to an emergency ( Figure 2).

The group has a structured programmatic handbook that details the purpose, mission, objectives, and rules of the group. It clarifies the structure of the group, the flow of information ( Figure 1), the frequency of the meetings, the responsibilities of each member, resources, and rules. Criteria to qualify as an author in the paper are also defined, as well as the position of these, ensuring the position of the student as the first author, as long as he/she fulfill the requirement.

Graduate (grad) students are defined as students who have finished medical school and must complete a one-year social service for the University before applying to post-graduate programs. This service may be in the community as a general physician, in the university hospital or medical school, or in research. Our grad students are usually GIA members who apply for research and can dedicate a full year to these activities. The student subgroup coordinator (SSC) is a GIA member that has been in the subgroup for at least 1 year and participates actively and will have the role of organizing and maintaining structure within each subgroup.

Formal training. Continuous education is made available to GIA members and professors. Every spring semester, a critical reading and writing course is organized in which research experts are invited to the sessions to teach and elaborate workshops for both students and professors, to improve their skills in research, critical thinking, problem-solving, manuscript writing, oral & written presentations, among others. Every fall semester, the mentors of each subgroup organize a workshop of the area of interest for the graduate and undergraduate members ( Table 1).

**Table 1.** GIA Subgroup Workshop examples

Subgroup	Workshop
General	Laboratory animal use and anesthesia
	Porcine skin model for basic sutures
Vascular	Human umbilical cord micro-dissection
	Cadaveric hepatectomy and dissection
	Live large vessel ultrasound morphometries
	Murine model large vessel dissection
Bones & Joints	Cadaveric arthroscopy of knee and shoulder
	Cadaveric knee ligament dissection
	Orthopedic casts & splints
	Musculoskeletal assessment (joint range and motion)
Central Nervous System	Cadaveric craniocervical surgical approach
	Cadaveric cervical spine surgery
	Cadaveric lumbar disc surgery
Peripheral Nervous System	Live peripheral nerve ultrasound-guided identification and morphometries
	Murine model nerve dissection
Orbit & Content	Retinal examination and scan
	Cadaveric eye extraction
	Cadaveric corneal surgery
Reproductive System	Trans-abdominal pregnancy ultrasound
	Cesarean techniques and outcomes
Head & Neck	Live ear & nose examination
	Rhinoplasty techniques
Medical Education	3D printing used for education
	Measuring methods and tools for surveying

The time invested varies for each role. Each student will spend various amounts of time with their respective SSC, grad student, and mentor, depending on the stage of their research. Each semester, 16 one-hour sessions are scheduled per subgroup. Many of the research skills are nurtured by discussing published papers, guiding proposed research ideas, and practicing keynotes for academic meetings, not only by the students but also by the professors. The mentors guide the session with students and provide clinical relevance to the morphological studies. Medical residents from different specialties rarely assist, so these work directly with mentors and 1 or 2 students outside the meetings ( Figure 2). Mentoring is also developed between younger and older students, as well as with the grad students. Aid is providing throughout all stages, from writing the protocol, submitting it to the ethics and research committees, executing the projects, data analysis, and publication.

Social gathering. Psychosocial activities are nurtured within the clubs. At the end of each semester, informal social gatherings with food are organized by the mentors of each subgroup. The students decide on an attire (e.g. formal, color of shirt or tie, lab coat, scrubs) and subgroup pictures are taken. Other social affairs are organized by the subgroups. During and after these events is when many of the members seek out mentors for advice, both personal and professional. It also allows for many of these mentor-mentee relationships to evolve into friendships, primarily amongst the younger mentors.

**Figure 3.**
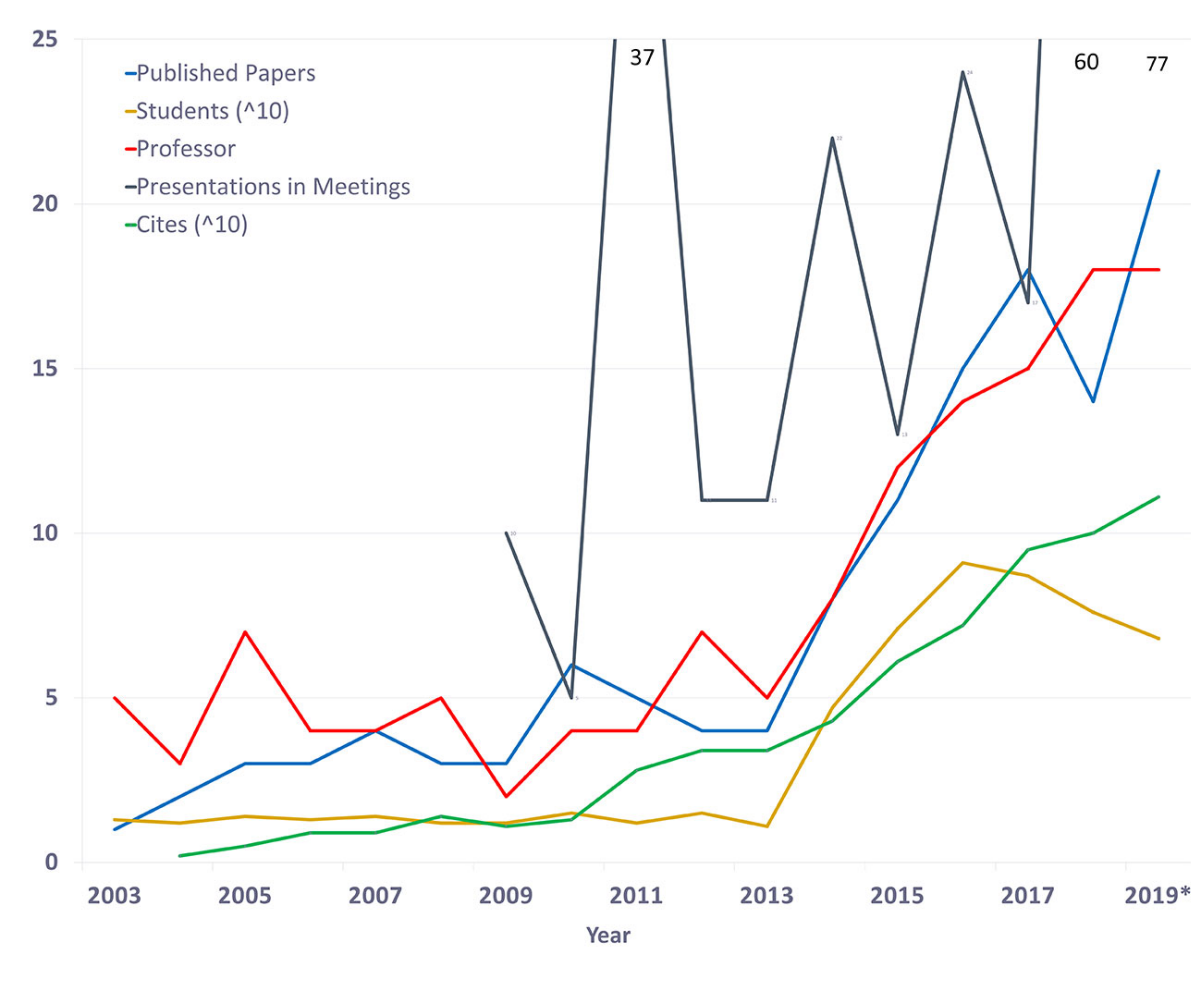
Anatomy Research Group Output.

The mentors are the voussoirs that maintain the structure of GIA. They are mixed between junior and senior faculty, although it is noteworthy that most of them are clinicians with a scientific focus, with few pure morphologists. Thirteen of the 18 professors currently have a Ph.D. in science or medicine, and four are currently enrolled in a Ph.D. program. We maintain a ratio of 2 to 4 mentors for each 8 to 12 student subgroup. Like most programs, the work is altruistic, as none receive direct financial remuneration. However, those with tenure, their participation, and productivity is evaluated by the University, for salary stimulus. We report the consistency, productivity, and evolution of GIA during a 16 years ( Figure 3). Data from 2019 includes up to the end of October 2019. Published papers for 2019 include 14 published/accepted papers and 7 under-review in indexed, peer-reviewed journals.

Published papers based on original scientific work, reviews, and letters to the editor. Results of 2019 include 14 published papers and 7 under-review (up to the end of October 2019). The number of students was expressed as a mean per year. Cites estimated according to google scholar analytics using Dr. Rodrigo Elizondo-Omaña’s profile (end of October 2019).

## Discussion

The model has proven to be effective over time with an increase in productivity (Morales-Avalos, Elizondo-Omana and Guzman-Lopez, 2014; Quiroga-Garza, Elizondo-Omana and Guzman-Lopez, 2018). The group maintains a low mentor-mentee ratio and activities are voluntary. Both professors and students integrated into the group by will, without scholarly pressure, obligation, or GPA exclusion ( Pololi and Knight, 2005; Desai *et al*., 2008; Wright, Titus and Cornelison, 2008). This has created a stress-free environment that the authors believe has also avoided misconduct ( Wright, Titus and Cornelison, 2008; Tan *et al*., 2018). During our 16-year experience, under supervision and with internal audits, there have not been any reports of conflict, misconduct, falsification of data, or unethical work. This may be due to the environment created in which mentors lead by example ( Wright, Titus and Cornelison, 2008), participate closely with students, the absence of the pressure of having to publish within a certain time period, publishing to graduate, or having to justify grants. Although some projects are grant-funded, students are not pressured with this. Most studies are morphological with clinical relevance, educational, and some experimental. Mentors are motived, also easily available for students outside meetings, and communicate regularly with them during the week by texting. Having the liberty to choose the mentor with whom the student wants to work with or working with more than one, also allows for more compatibility, and sharing of mutual interest ( Pololi *et al*., 2002; Hauer *et al*., 2005; Ratnapalan, 2010; Tan *et al*., 2018).

Mentors support, educate, and encourage students into academic activities. They promote scholarly values, scientific integrity, and ethical decision making. They become “intellectual parents” ( Chopin, 2002; Gershenfeld, 2014). Students slowly become proficient and fully capable researchers through training and experience not only within the group, but through exposure and active participation in presentations, meetings, and conferences ( Chopin, 2002; Brown, Daly and Leong, 2009).

Hierarchical ranks have been avoided in the group. Although there is an organigram, this is primarily to establish the flow of information and responsibility for all activities. The relationships between all members are more of colleagues ( Mullen, 2000; Pololi *et al*., 2002; Hauer *et al*., 2005; Feldman, Divoll and Rogan-Klyve, 2013; Morales-Avalos, Elizondo-Omana, Guzman-Lopez, 2014; Stevenson *et al*., 2017), although a sense of respect and admiration forms naturally from the student towards its mentor. The easy access to high ranking researchers/professors not only in official meetings but also through social gatherings creates a sense of belonging for the students ( Feldman, Divoll and Rogan-Klyve, 2013). Enforcing the rule of allowing the student to maintain the first author position when they play an important role in developing the idea (initiative) and carrying out the project (persistence), also adds to the sense of not only belonging as a co-researcher, but of ownership ( Pololi *et al*., 2002; Desai *et al*., 2008; Gershenfeld, 2014; Linn *et al*., 2015). The project was made possible by the student and should be cited with their family name, avoiding abuse of a mentor by usurping the work. A well-structured program creates a more effective group ( Pololi and Knight, 2005; Gershenfeld, 2014; Tan *et al*., 2018).

The collaboration was a basic element for the growth of the group. The inclusion of professors from different departments has broadened the group’s perspective on research, allows more discussion, feedback, and consistency, avoiding canceling meetings due to unexpected changes in agenda. This structure allowed for academic flexibility, creating quality in the time invested. The mentors do not have time protection for research, they are clinicians, a problem solved by the collaborative mentoring, and an example to students of the mentor’s dedication ( Mullen, 2000; Pololi *et al*., 2002; Feldman, Divoll and Rogan-Klyve, 2013; Menon, Muraleedharan and Bhat, 2016; Schwartz *et al*., 2016; Tan *et al*., 2018). The collaboration was extended to several levels. Mentors were included in the diverse workshops offered by the group and other mentors in topics of interest, improving research and writing skills. Many Universities lack this formal education and training for mentors ( Pololi *et al*., 2002; Wright, Titus and Cornelison, 2008; Amgad *et al*., 2015; Funston, 2015; Linn *et al*., 2015; Aguilar, 2016; Menon, Muraleedharan and Bhat, 2016; O’Loughlin, Husmann and Brokaw, 2018; Quispe-Juli *et al*., 2019). The collaboration was also extended to the publication, granting authorship to those who participated and met the criteria of such position. The most active members were primarily junior and young faculty; however, the success of the group has constantly attracted senior professors, inducing more innovative ideas with higher impact ( Mullen, 2000; Pololi *et al*., 2002; van Eyk *et al*., 2010; Feldman, Divoll and Rogan-Klyve, 2013; Palmer *et al*., 2015; Quiroga-Garza, Elizondo-Omana and Guzman-Lopez, 2018; Havard and Baker, 2018).

Many authors discuss different mentoring and research group strategies; however, few include results and long-term output. Although it has only been implemented in one institution, GIA demonstrates longitudinal evidence of persistence through a tightly organized, well-structured, formal research group model that includes members from different levels, and academic productivity (published papers and participation in meetings) as an outcome. As Figure 2 demonstrates, it takes 5 to 10 years to clearly evidence results. It provides a solution to the lack of mentor in research and low undergraduate student participation in many medical schools ( Pololi and Knight, 2005; Feldman, Divoll and Rogan-Klyve, 2013; Linn *et al*., 2015; Menon, Muraleedharan and Bhat, 2016; Linn *et al*., 2015; Tan *et al*., 2015; O’Loughlin, Husmann and Brokaw, 2018; Quispe-Juli *et al*., 2019).

The GIA model can be an example of effective mentoring in a medical school than can be applied in low, middle, and high-income countries. However, there are several limitations to the model. Although satisfaction amongst mentors and mentees has been noticeable, as both constantly speak well of the group, and boost recruitment, the authors have not formally evaluated this, nor how it may aid in avoiding burn-out amongst professors or promote future scientific specialists. Willingness to participate is evident, as it is an extracurricular activity, meaning they are there, because they want to be there, however, assessment of several parameters (satisfaction, obstacles, time, pressure, respect, availability, compatibility, etc.) should be performed in the near future to search for areas of improvement ( Menon, Muraleedharan and Bhat, 2016; Tan *et al*., 2018). There have also been several undergraduate students who drop-out of the group, desert their projects, or lack productivity. Although this has been the minority, the exact frequency of this has not been evaluated, nor the motive behind it. Future studies will be performed to evaluate this. Formal surveying of career paths and involvement in research of prior GIA members is lacking, yet, the majority still follow a clinical residency. However, many of those who were prior research grad students and SSC, are actively publishing in their fields.

Starting was the challenge, perseverance was our objective, diversity our strength, belonging our success. “Alone we can do so little, together we can do so much” - Helen Keller

## Take Home Messages

• Collaborative mentoring between mentors is effective, avoiding a one-individual dependence.
• Research involvement should be voluntary and motivational, creating a favorable mentor-mentee relationship
• Students should be encouraged to present projects and advances in diverse meetings, and be included in publications, as co-authors to motivate further participation.
• A formalized, well-structured research group succeeds in longitudinal output.

## Notes On Contributors

RODRIGO ENRIQUE ELIZONDO-OMAÑA, M.D., Ph.D., is a full-time professor and general coordinator in the Human Anatomy Department at the School of Medicine, Universidad Autónoma de Nuevo Leon, Monterrey, Nuevo León, México. He teaches human gross anatomy to graduate and undergraduate students and is a member of the National Research System (SNI) and anatomy research group.

PABLO PATRICIO ZARATE-GARZA, M.D., Is a full-time professor in the Human Anatomy Department at the School of Medicine, Universidad Autónoma de Nuevo León, Monterrey, México. He teaches human gross anatomy to graduate and undergraduate students and is a member of the anatomy research group.

GUILLERMO JACOBO-BACA, M.D., Is a full-time professor and academic coordinator in the Human Anatomy Department at the School of Medicine, Universidad Autónoma de Nuevo León, Monterrey, México. He teaches human gross anatomy to graduate and undergraduate students and is a member of the anatomy research group.

YOLANDA SALINAS-ALVAREZ, M.D., M.Sc., Is an associate professor in the Human Anatomy Department at the School of Medicine, Universidad Autónoma de Nuevo León, Monterrey, México. She teaches human gross anatomy to undergraduate students and is a member of the anatomy research group.

BERNARDO ALFONSO FERNANDEZ-RODARTE, M.D., Is a full-time professor in the Human Anatomy Department at the School of Medicine, Universidad Autónoma de Nuevo León, Monterrey, México. He teaches human gross anatomy to graduate and undergraduate students and is a member of the anatomy research group.

JAVIER HUMBERTO MARTINEZ-GARZA, M.D., Ph.D., Is a full-time professor in the Human Anatomy Department at the School of Medicine, Universidad Autónoma de Nuevo León, Monterrey, México. He teaches human gross anatomy to graduate and undergraduate students and is a member of the anatomy research group.

ALEJANDRO QUIROGA-GARZA, M.D., M.Surg., Ph.D., Is a full-time professor and research coordinator of the anatomy research group in the Human Anatomy Department at the School of Medicine, Universidad Autónoma de Nuevo León, Monterrey, México. He teaches human gross anatomy to graduate and undergraduate students, and is a member of the National Research System (SNI).

SANTOS GUZMAN-LOPEZ, M.D., Ph.D., is Chair of the Human Anatomy Department at the School of Medicine, Universidad Autónoma de Nuevo León, Monterrey, Nuevo León, México. He teaches human gross anatomy to graduate and undergraduate students and is a and is a member of the National Research System (SNI) and anatomy research group.

## Declarations

The author has declared that there are no conflicts of interest.

## Ethics Statement

As a descriptive manuscript, no ethics approval was required, however, all research publications related to the group structure and activities were approved by the University’s ethics and research committees with the registration number PI19-00262.

## External Funding

This article has not had any External Funding
