# Supplementary material for: Collaborative mentoring for effective medical research groups
Source: MedEdPublish (2016). 2019 Nov 26;8:214. [Version 1] doi: 10.15694/mep.2019.000214.1 (PMC10712473; doi:10.15694/mep.2019.000214.1)
Supplement: Peer review history

# Reviewer report: Reviewer response for version 1

**DOI:** 10.21956/mep.19944.r30281

**Published:** 2019-12-08

**Authors:** PATRICIA CURY

**Affiliations:** 1 FACERES

This review has been migrated. The reviewer awarded 5 stars out of 5 This is a very interesting paper and a very original model mixing mentoring and anatômicas-clínical research ! And all of this with voluntary work! I just wonder how was the percentage of faculty members and students participate in this program?

Reviewer Expertise:

NA

No decision status is available

# Reviewer report: Reviewer response for version 1

**DOI:** 10.21956/mep.19944.r30280

**Published:** 2019-12-07

**Authors:** Helena Filipe

**Affiliations:** 1 Hospital of the Armed Forces/PL-EMGFA

This review has been migrated. The reviewer awarded 5 stars out of 5 It was a pleasure reading this article focusing on the benefits and challenges of mentorship. Authors highlighted the creation, implementation and evaluation of a formal mentorship program within the medical education context.The article reads well and describes clearly the motivation to pursue a well –structured mentorship program and the staged process undertaken towards its implementation, pilot and progress.The introduction frames the importance of mentorship with an emphasis for a statement that particularly defines what mentorship entails: “When two or more individuals from different educational levels/expertise come together to work on a common objective, a certain grade of mentoring will naturally evolve” The article offers rich and consistent evidence to sustain the development, implementation and regulation of the mentoring model in appreciation.Authors provide a stepwise, full and clear description as how they created and progressed from a” loosely organized group with a low frequency of meetings and a low number of mentors, to a well-structured, tightly organized … collaborative mentoring … group during a four- stage process.” The writing is fluid and translates well the excitement and engagement of all involved in this increasingly rich and multidisciplinary community of learning. The community is well characterized and their experience shared: “To construct an effective research group, three elements were essential: student initiative, persistence, and collaborative mentoring.” The bonds created among members of this community in which one can be simultaneously mentor and mentee weave a fantastic network to collaborative create positive human relationships and develop research. The output of this project, with an upshot after 2013, is remarkable.Authors discuss thoughtfully their model, progression and results attained highlighting attributes as a “low mentor-mentee ratio”, “activities are voluntary”, “professors and students integrated into the group by will”, “without scholarly pressure, obligation, or GPA exclusion”, a “stress-free environment”., “Hierarchical ranks have been avoided in the group”. Throughout the narrative the reader is offered pearls of experience, such as holding internal audits, self-regulation, principles of authorship initiative, perseverance, ownership, inclusiveness, collaboration, independence and responsibility, wide perspective and interdisciplinary, positive relationships, engagement, effectiveness and accomplishment, teamwork, diverse skills and knowledge. Authors could find no better end but Helen Keller’s saying “Alone we can do so little, together we can do so much”Well done!

Reviewer Expertise:

NA

No decision status is available

# Reviewer report: Reviewer response for version 1

**DOI:** 10.21956/mep.19944.r30282

**Published:** 2019-11-28

**Authors:** P Ravi Shankar

**Affiliations:** 1 American International Medical University

This review has been migrated. The reviewer awarded 4 stars out of 5 I enjoyed reading this description of collaborative research projects primarily in the Department of Anatomy at a medical school in Mexico. The authors have described collaborative research projects among undergraduate, graduate, masters, residency students and faculty members. The research group has been in place for over 16 years and there has been a steady increase in participation and in research output. The quality of written English is mostly very good but can benefit from editing in a few areas. I am interested in knowing the research group’s criteria for determining that a student is eligible to be the first author of a paper. I would also be interested in a few examples of published research work by the group. I did not fully understand figures 1 and 2. A brief description of the different subdivisions of the Anatomy department within which the research group is cited will be helpful. I would like to congratulate the authors for their long-term commitment to research and wish them luck for the future.

Reviewer Expertise:

NA

No decision status is available
